# Validation of a high resolution NGS method for detecting spinal muscular atrophy carriers among phase 3 participants in the 1000 Genomes Project

**DOI:** 10.1186/s12881-015-0246-2

**Published:** 2015-10-29

**Authors:** Jessica L. Larson, Ari J. Silver, Dalin Chan, Carlos Borroto, Brett Spurrier, Lee M. Silver

**Affiliations:** GenePeeks, Inc., Cambridge, MA USA; GenePeeks, Inc., New York, NY USA; Department of Molecular Biology and the Woodrow Wilson School of Public and International Affairs, Princeton University, Princeton, NJ USA

**Keywords:** 1000 Genomes Project, Carrier testing, DNA-seq, Exome-seq, Genetic testing, Next-generation sequencing (NGS), *SMN1*, *SMN2*, Spinal muscular atrophy (SMA)

## Abstract

**Background:**

Spinal muscular atrophy (SMA) is the most common pan-ethnic cause of early childhood death due to mutations in a single gene, *SMN1*. Most chromosome 5 homologs have a functional gene and dysfunctional copy, *SMN2*, with a single synonymous base substitution that results in faulty RNA splicing. However, the copy number of *SMN1* and *SMN2* is highly variable, and one in 60 adults worldwide are SMA carriers. Although population-wide screening is recommended, current SMA carrier tests have not been incorporated into targeted gene panels.

**Methods:**

Here we describe a novel computational protocol for determining SMA carrier status based solely on individual exome data. Our method utilizes a Bayesian hierarchical model to quantify an individual’s carrier probability given only his or her *SMN1* and *SMN2* reads at six loci of interest.

**Results:**

We find complete concordance with results obtained with the current qPCR-based testing standard in known SMA carriers and affecteds. We applied our protocol to the phase 3 cohort of the 1,000 Genomes Project and found carrier frequencies in multiple populations consistent with the present literature.

**Conclusion:**

Our process is a convenient, robust alternative to qPCR, which can easily be integrated into the analysis of large multi-gene NGS carrier screens.

**Electronic supplementary material:**

The online version of this article (doi:10.1186/s12881-015-0246-2) contains supplementary material, which is available to authorized users.

## Background

Spinal muscular atrophy (SMA) is a common autosomal recessive disorder affecting approximately 1/10,000 live births [1]. The disease results from the degeneration of spinal cord motor neurons, which leads to the progressive weakness and deterioration of skeletal muscle, and eventually paralysis and death [2]. SMA is categorized into four clinical types based on disease manifestations and age of onset [3]. Type I (OMIM: 253300) patients have severe muscle weakness within the first three months of life; death usually occurs within the first two years. Patients with Type II SMA (OMIM: 253550) are able to sit, but cannot stand or walk; they typically survive beyond four years. Type III SMA (OMIM: 253400) is a milder form of SMA; these patients are diagnosed later in their youth and can walk unaided [2, 3]. The mildest adult-onset form of SMA (Type IV, OMIM: 271150) is found in patients who can walk into adulthood [2].

**Fig. 1:**
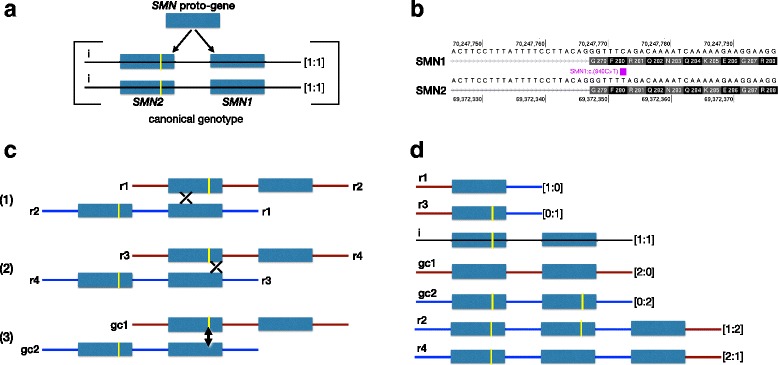
Evolution of the human *SMN* locus. **a**. The human *SMN1* and *SMN2* genes were derived by duplication of a *proto-SMN* gene after the human-chimpanzee split. The yellow bar represents the only functional base change that distinguishes *SMN2* from *SMN1* (on chromosome 5 at position 69,372,353 in the GRCh37/hg19 reference genome) which is signified on the canonical transcript position as c.840C > T. The copy number of each gene on a single chromosome is indicated in the bracket and colon formulation [*SMN1*:*SMN2*]. A canonical *SMN* chromosomal locus consists of one copy of each gene in the centromere-telomere order *SMN2*-*SMN1*. A canonical homozygous genotype is represented as [1:1]/[1:1]. **b**. Comparison of *SMN1* and *SMN2* sequences on either side of the gene-defining c.840C > T base difference. **c**. Three categories of interlocus homologous recombination between *SMN1* and *SMN2* generate copy number variants of the *SMN* locus. 1. Recombination and exchange on the centromeric side of the *SMN2*-defining base; 2. recombination and exchange on the centromeric side of the *SMN2*-defining base; 3. interlocus gene conversion across the *SMN2*-defining base region, indicated with the double-arrowed line. **d**. Each recombination event produces two variant *SMN* chromosome types which are cross-referenced from panel c. Variant chromosomes are ordered by total *SMN* copy number

All autosomal recessive forms of SMA disease are caused by variant forms of the SMN locus on chromosome 5 (chr5), which ordinarily contains two nearly identical copies of a gene encoding the survival of motor neuron gene product [4]. The two gene copies, referred to as *SMN1* and *SMN2*, were derived through a recent duplication event along the human lineage (Fig. 1) [5]. They have identical exon-intron organizations and the potential for the same gene product.

*SMN1* and *SMN2* are typically distinguished by a handful of single nucleotide differences, only one of which has an impact on the corresponding polypeptide [2, 5–9]. This single functional difference occurs at the sixth base of the eighth exon (referred to traditionally as exon 7) (840 C > T). The *SMN2* base (T) is synonymous in terms of amino acid coding, but causes a rate of exon-skipping of 50-90 % (depending on the tissue analyzed) and corresponding reduction of gene functionality (Fig. 1b, Additional file 1: Figure S1a) [10–13].

Variant forms of the *SMN* locus are produced at a frequency of approximately 1 in 10,000 by intergenic meiotic recombination between homologous regions within and around *SMN1* and *SMN2* (Fig. 1c). Unequal crossing-over generates two chromosomal products containing one gene or three genes, respectively. Depending on the location of the cross-over event, the single gene chromosome will carry either *SMN1* or *SMN2*, and the three gene chromosome will have a 1:2 or 2:1 *SMN1*:*SMN2* ratio (Fig. 1d). Alternatively, at a much lower frequency, heteroduplex formation across the gene-defining *SMN* location will resolve without crossing over, leading to gene conversion. Gene conversion does not alter copy number but can result in two copies of either *SMN1* or *SMN2*. Due to the high degree of repetitive sequence in *SMN1*, intragenic recombination is also quite likely and can lead to deleterious variations [14].

As a result of recombination in the *SMN* locus, a large degree of variability in *SMN1* and *SMN2* copy number exists in the global human population [14]. Up to 15 % of individuals lack *SMN2* completely, while others can have three or more copies of this gene [10, 14–17]. However, all variant chromosomes formed by intergenic recombination must carry at least one *SMN* gene that may be *SMN1* or *SMN2*.

Carrier frequency for SMA ranges from about 1/47 in European populations to 1/72 in African Americans [10, 18, 19]. Approximately 95 % of SMA cases are a consequence of *SMN1* gene transformation by unequal crossing over or gene conversion [20]. Most of the remaining SMA affected patients are compound heterozygotes with intragenic mutations in their single remaining copy of *SMN1* [4, 10, 15].

Prompted by the severity of SMA and its high pan-ethnic incidence rate, the American College of Medical Genetics and Genomics has recommended universal SMA carrier testing in order to identify couples at risk of conceiving an SMA affected child [21]. The conventional SMA screening protocol involves some form of quantitative polymerase chain reaction (qPCR) directly, or in combination with multiplex ligation-dependent probe amplification (MLPA), TaqMan, restriction fragment length polymorphism, denaturing high-performance liquid chromatography, or direct (Sanger) sequencing [15, 22–27] (see [28] for a review). qPCR primers are designed specifically to amplify segments of exon 7 containing the *SMN1*-defining sequence. The copy number of *SMN1* is calculated by comparing its cycle threshold directly to that of a control gene(s). One of the most robust methods to detect SMA carriers is MLPA, a qPCR based method that utilizes fragment fluorescence intensity to determine *SMN1* and *SMN2* copy number [23, 29, 30].

While existing qPCR approaches are accurate on a case-by-case basis, none of the current processes can be incorporated into cost-effective NGS screens for the simultaneous detection of carrier status at hundreds of genes [31]. In this paper we present a novel Bayesian analysis protocol to determine the probability that an individual is an SMA carrier given only his or her sequencing coverage at a few sites of interest. We applied our technique to known SMA carriers and over fifty samples with unknown genotypes. Our NGS method accurately predicted the status of known carriers, and provides output as a continuous likelihood, rather than a binary scale, for each individual. We applied our algorithm to determine the carrier status of individuals from the Phase 3 release of the 1000 Genomes Project [32] to demonstrate its effectiveness in a large diverse sequencing study where qPCR is not a reasonable alternative. Our statistical approach is a useful method for determining SMA carrier status from only DNA-sequencing data and should be part of a large NGS carrier-testing platform.

## Methods

### Sample collection and initial processing

We collected semen or saliva samples from volunteers. We anonymized sample names to conceal personal identification information.

Saliva samples were collected with the Oragene Dx (ODG-510) collection kit (DNA Genotek, Kanata Ontario, Canada). Genomic DNA was extracted from saliva samples using the prepIT L2P (DNA Genotek, Kanata Ontario, Canada) reagents per the manufacturer’s instructions. Semen samples were immediately cryopreserved at −190 °C after collection. DNA was extracted from the semen samples using the MasterPure Complete DNA & RNA Isolation Kit (Epicenter, Madison WI, USA) according to the manufacturer’s specifications. Genomic DNA from both saliva and semen were re-purified using the Genomic DNA Clean and Concentrator-5 Kit (Zymo Research, Irvine CA, USA) according to the manufacturer’s protocol.

The integrity for both the saliva- and semen-extracted DNA was verified by electrophoresis in 2 % agarose gels. DNA concentration and quality were measured with the Qubit dsDNA HS Assay kit (Life Technologies, Grand Island NY, USA) and the NanoDrop 8000 spectrophotometer (NanoDrop, Wilmington DE, USA). NanoDrop OD ratios above 1.7 for A260/280 and above 1.7 for A260/230 indicate high purity of samples. Final DNA concentration for each sample was adjusted to 4 μg/ml, a sufficient amount to obtain at least 2 μg of genomic DNA per sample. Final DNA samples were aliquoted and stored at −20 °C prior to use. Genomic DNA samples that did not meet OD ratios above 1.7 and had DNA content below 100ng were re-collected or removed from NGS processing.

Additionally, 2,532 aligned BAM files from the Phase 3 of the 1000 Genomes Project (1KG3) were downloaded for subsequent processing (ftp://ftp-trace.ncbi.nih.gov/1000genomes/ftp/) [32]. We removed 31 samples that were classified as related by the 1000 Genomes Project Consortium.

### Ethics, consent and permissions

The GenePeeks Research Ethics Committee approved research use of saliva and semen samples. All samples were previously collected from volunteers consenting to use their sample and derivative information for additional genetic testing or future research. We obtained written consent from all participants.

### Consent to publish

Each volunteer participant gave written consent to publish his or her individual data.

### Sequencing

The Illumina TruSight Inherited Disease panel (Illumina, San Diego CA, USA) was chosen to sequence samples. The panel contains 552 disease-associated genes, including *SMN1* and *SMN2*. A total of 150 ng of genomic DNA collected from saliva, semen, and Coriell Institute DNA samples were prepared with the panel according to the manufacturer’s protocol. Pooled libraries were size selected on the Pippin Prep (Sage Sciences, Beverly MA, USA) for 300-700bp and were sequenced on the Illumina MiSeq at 2x 300 cycles.

### Bioinformatics processing

We used the Burrows-Wheeler Alignment tool (BWA MEM) [33] to align the sequence reads to the human reference genome, GRCh37/hg19 (downloaded from the Genome Analysis Toolkit (GATK) version 2.8), creating a single aligned BAM file for each individual saliva, sperm, and Coriell sample. Potential PCR duplicate reads were marked by running the aligned subject data through the Picard MarkDuplicates tool (http://broadinstitute.github.io/picard/). We then performed local realignment for each sample by using the GATK IndelRealigner tool followed by recalibrating base qualities using the GATK BaseRecalibration tool, both according to GATK recommended best practices [34].

The average gene coverage for individual genes was determined for each saliva, sperm, Coriell, and 1KG3 sample by running the resulting BAM files through GATK’s DepthOfCoverage analysis tool. Total coverage for three *SMN1* loci on chromosome 5 (chr5:70,247,724, chr5:70,247,773, and chr5:70,247,921) and three *SMN2* loci (chr5:69,372,304, chr5:69,372,353, and chr5:69,372,501) was parsed from the output data from the DepthOfCoverage tool.

### MLPA

Copy number status was determined by Multiplex ligation-dependent probe amplification (MLPA) [23]. We used 200ng per sample reaction of genomic DNA from human saliva, semen, and Coriell Institute DNA samples with the SALSA MLPA SMA P060 probe mix kit (MRC-Holland, Netherlands) according to the manufacturer’s protocol (Additional file 2: Table S1). The MLPA PCR products were separated and captured for probe fluorescence intensity by capillary electrophoresis on the ABI3730XL (Applied Biosystems, Foster City CA, USA). Raw data files were analyzed for copy number status on the Coffaylser. NET software (MRC-Holland, Netherlands). Two Coriell Institute samples with 1 copy of *SMN1* and 2 copies of *SMN2* were chosen as positive controls for MLPA. We chose three reference controls that have 2 copies of *SMN1* and 2 copies of *SMN2* from a previous MLPA experiment using SD019 Reference DNA (MRC-Holland, Netherlands) as the SMA normal reference control. Samples were run in duplicate; final *SMN1* to reference ratios reported in the main text and tables represent averages of these values.

### *SMN1* reads at loci of interest

We consider *N* subjects. Let *D*_*bi*_ *= 0, 1, 2, …,r*_*bi*_ be the number of reads that align to *SMN1* in the *i*th subject *(i = 1,2,…,N*) at chr5:70,247,773, where *r*_*bi*_ is the total number of reads aligned to the *SMN1* region (chr5:70,247,773) and the analogous *SMN2* region (chr5:69,372,353). Note that these two loci, denoted with the letter ‘*b*’ and located in exon 7 of their respective genes, correspond to the only coding differences between *SMN1* and *SMN2*. We also examined two intronic loci on either side of exon 7 symbolized by *a* (chr5:70,247,724 in *SMN1* and chr5:69,372,304 in *SMN2*) and *c* (chr5:70,247,921 in *SMN1* and chr5:69,372,501 in *SMN2*).

We use π_i_ to represent the probability that a *SMN1* or *SMN2* (denoted as *SMN*) read is actually from *SMN1* in the *i*th subject. We define ${\widehat{\pi}}_{bi}$ as the observed proportion of *SMN* reads that align to *SMN1* in exon 7 (i.e., ${\widehat{\pi}}_{bi}={D}_{bi}/{r}_{bi}$), and calculated ${\widehat{\pi}}_{ai}$ and ${\widehat{\pi}}_{ci}$ in a parallel manner. Our observed proportion of *SMN1* reads is ${\widehat{\pi}}_i={D}_i/{r}_i$*,* where, for most samples, *D*_*i*_ is the total number of *SMN1* reads and *r*_*i*_ is the total number of *SMN* reads at our three loci of interest (i.e., *D*_*i*_ = *D*_*ai*_ + *D*_*bi*_ + *D*_*ci*_ and *r*_*i*_ = *r*_*ai*_ + *r*_*bi*_ + *r*_*ci*_). If $\left|{\widehat{\pi}}_{bi}-{\widehat{\pi}}_{ai}\right|>\in$ or $\left|{\widehat{\pi}}_{bi}-{\widehat{\pi}}_{ci}\right|>\in$, for some ∈ > 0 (∈ = 0.10, here), then the so-called ∈ condition is not met and we let *D*_i_ *= D*_*bi*_ and *r*_i_ *= r*_*bi*_.

### Scaled *SMN1* reads

To further account for *SMN1* copy number, we considered *K* “housekeeping” or control genes (*k = 1, 2,…, K)* and calculate *z*_*ki*_ *= (c*_*1i*_ *+ c*_*2i*_*)/H*_*ki*_, where *c*_*1i*_ is the average coverage for the *SMN1* gene region, *c*_*2i*_ is the average coverage for *SMN2,* and *H*_*ki*_ is the average coverage for gene *k* in the *i*th subject.

For each person, we calculated a weighted average of the coverage of *SMN1* to our K housekeeping genes: ${\widehat{\theta}}_i=\frac{{\displaystyle {\sum}_{k=1}^K{z}_{ki}/{\overline{z}}_k}}{K}$, where ${\overline{z}}_k=\frac{{\displaystyle {\sum}_{i=1}^N{z}_{ki}}}{N}$ and *N* is the total number of subjects. For conservatism, any values of ${\widehat{\theta}}_i>1.0$ are set to 1.0 so that our scaling factor has a ceiling of 1.00 (i.e., ${\widehat{\theta}}_i\in \left[0,1\right]$).

Selecting housekeeping genes representative of genome-wide copy-number is nontrivial. We wanted to only include genes that have sufficiently high coverage in the majority of subjects. Genes with low coverage (lower than the 5th percentile in at least 10 % of subjects) were not considered. Those that passed this coverage filter were then selected for one of two properties: (1) low variability in average coverage across all samples or (2) low variability in *z*_*ki*_ across all samples. To account for differences in scale, we used the coefficient of variation across all samples ($\widehat{\theta}/\widehat{\mu}$) to rank the variability of each gene. We chose the top ten genes according to properties (1) and (2) for a total of *K = 20* control genes. Unlike other copy number determining algorithms, our housekeeping genes do not necessarily need to be 2-copy across all samples, but they do need consistent coverage relative to *SMN.*

We use ${\widehat{\theta}}_i$ to scale our observed *SMN1* reads to ${D_i}^{\prime }={\widehat{\theta}}_i{D}_i$ and let ${{\widehat{\pi}}_i}^{\prime }={D_i}^{\prime }/{r}_i$ represent our scaled estimate of *π*_*i*_*.* In this way, we account for the number of *SMN* reads relative to low variability regions.

### Bayesian hierarchical model

Assuming that we align to either a *SMN1* or *SMN2* at our polymorphic sites, the (scaled) number of *SMN1* reads is binomially distributed: ${D_i}^{\prime }=\widehat{\theta}{D}_i\sim Bin\left({r}_i,{\pi}_i\right)$, where π_i_ is the probability that a *SMN* read is actually from *SMN1* in the *i*th subject. Thus, $$ P\left({D_i}^{\prime }={d}_i\left|{r}_i,{\pi}_i\right.\right)=\left(\begin{array}{c}\hfill {r}_i\hfill \\ {}\hfill {d}_i\hfill \end{array}\right){\pi}_i^{d_i}{\left(1-{\pi}_i\right)}^{r_i-{d}_i} $$. The binomial distribution allows us to model the reads in our dataset; however, we are more interested in making inference about *π*_*i*_. In particular, we want to know

$$ \begin{array}{c}\mathrm{P}\left(\mathrm{subject}\ \mathrm{is}\ \mathrm{a}\ \mathrm{c}\mathrm{a}\mathrm{rrier}\Big|\mathrm{sequencing}\ \mathrm{data}\right) = \mathrm{P}\left(\mathrm{ratio}\ \mathrm{o}\mathrm{f}\kern0.5em SMN 1\kern0.5em \mathrm{reads}\ \mathrm{t}\mathrm{o}\kern0.5em SMN 2\kern0.5em \mathrm{reads}\ \mathrm{is}\ 1:2,\ 1:3,\ \mathrm{e}\mathrm{t}\mathrm{c}.\right)\\ {} = \mathrm{P}\left(\mathrm{proportion}\ \mathrm{o}\mathrm{f}\ SMN 1\kern0.5em \mathrm{reads}\ \mathrm{is}\ 1/3,\ 1/4,\ \mathrm{e}\mathrm{t}\mathrm{c}.\right)\\ {} = P\left({\pi}_i\le\ 1/3\Big|{D}_i^{\prime },\ {r}_i\right).\end{array} $$

To this end, we use a Bayesian hierarchical model and assume a (conjugate) prior for *π*_*i*_: *π*_*i*_ *∼ Beta(α, β)*. Thus, the posterior distribution for *π*_*i*_ is also a beta distribution:

$$ {\pi}_i\left|{D_i}^{\prime },{r}_i\right.\sim Beta\left(\alpha +{D_i}^{\prime },{r}_i-{D_i}^{\prime }+\beta \right),\forall i. $$

We calculate *P*(*π*_*i*_ ≤ 1/3|*D*_*i*_′, *r*_*i*_) directly via the cumulative distribution function of our posterior beta distribution. In order to conservatively capture all potential carriers, we allow a 5 % “Type I error” and present carrier probability results calculated As *P*(*π*_*i*_ ≤ 0.38|*D*_*i*_′, *r*_*i*_) rather than *P*(*π*_*i*_ ≤ 1/3|*D*_*i*_′, *r*_*i*_). We considered both a uniform prior (α = β = 1) and Jeffreys noninformative prior (α = β =1/2).

This method can easily be extended to the case where our loci are not biallelic with a multinomial-Dirichlet Bayesian hierarchical model.

### Sample categorization

We classified each sample into one of three categories based on its corresponding credible interval. Individuals with 95 % credible intervals for *π* entirely below 0.38 are “likely” carriers; those with intervals that span 0.38 are “possibly” carriers. All others are “unlikely” carriers. Our posterior carrier probability (and corresponding credible intervals) is calculated to determine the probability that an individual has a true *π* ≤ 0.38 given the data. Unlike frequentist confidence intervals, there is a 95 % chance that the true π is in the bounds of the given 95 % credible interval. Thus, if an individual’s credible interval contains 0.38, it is literally possible that he or she is an SMA carrier. Note that because our final posterior carrier probability is continuous on the [0,1] scale, this metric is more meaningful than the discrete “likely”, “possible”, and “unlikely” carrier categorizations. We recommend that clinical users of our method verify results by MLPA for “possible” carriers in the clinic.

### Theoretical results

In theory, the copy number ratio of *SMN1*, *SMN2*, and each housekeeping region are consistent among all individuals with two copies of *SMN1*, *SMN2*. Thus, normal SMA copy number individuals will have a ${z}_{ki}/{\overline{z}}_k=1$ and a calculated weighted average ${\widehat{\theta}}_i=1$ (Additional file 3: Table S2). Those with fewer than two copies of *SMN1* and/or *SMN2* will have ${\widehat{\theta}}_i<1$ (Additional file 3: Table S2). Observed carrier frequencies were taken from [14] and [16].

To determine the relationship between coverage and carrier probability, we obtained 1,000 random realizations of *D|r*,π and calculated the average *P*(*π* ≤ 0.38|*D*, *r*) for a given value of *r* and π.

## Results

### Volunteer, Coriell, and 1000 Genomes Samples sequenced for carrier testing

To determine the accuracy of our method, we collected and sequenced DNA from 56 healthy volunteers and 15 samples from the Coriell Institute. The volunteer samples were collected via saliva (n_saliva_ = 47) or semen (n_semen_ = 7); there was no difference in their average sequencing coverage (*p*-value = 0.195). The Coriell cohort included nine positive controls: five individuals affected with SMA and four SMA carriers. We also analyzed six diagnosed non-SMA diseased control Coriell samples: two normal healthy controls (NHC), two with amyotrophic lateral sclerosis (ALS), one with Duchenne muscular dystrophy (DMD), and one with X-linked SMA (SMAX1). While four of these individuals are affected with a neuromuscular disease having a notable genetic component, they are only as likely as a randomly selected individual to be an SMA carrier. Additionally, we processed the raw sequencing data from 2,501 unrelated members of the 1000 Genomes Project Phase 3 (1KG3) release; the carrier status of these samples was completely unknown.

### Novel sequencing-based SMA carrier detection method

We developed a Bayesian hierarchical model to quantify the probability that an individual has at most one copy of *SMN1* given his or her distribution of aligned DNA-seq reads to *SMN1* at three nucleotide differences that distinguish canonical *SMN1* and *SMN2* (Methods, Additional file 1: Figure S1b). Briefly, we assume that the number of reads aligning to the loci in *SMN1* and *SMN2* can be modeled by a binomial distribution with a fixed number of total reads and probability, π, that a read aligned to this region is actually from *SMN1*. We found almost no difference between the outcomes of two uninformative priors for our model (Additional file 4: Figure S2) and proceeded with Jeffreys conjugate prior.

Given our sequencing data and prior, the posterior of our parameter of interest (π) follows a closed-form beta distribution, making corresponding carrier probabilities computationally easy to calculate (Methods). The chance of being a carrier is inversely proportional to the number of reads aligned to *SMN1*; subjects with an observed estimate of π (denoted as $\widehat{\pi}$) less than 1/3 have high carrier probabilities (Additional file 3: Table S2).

**Fig. 2:**
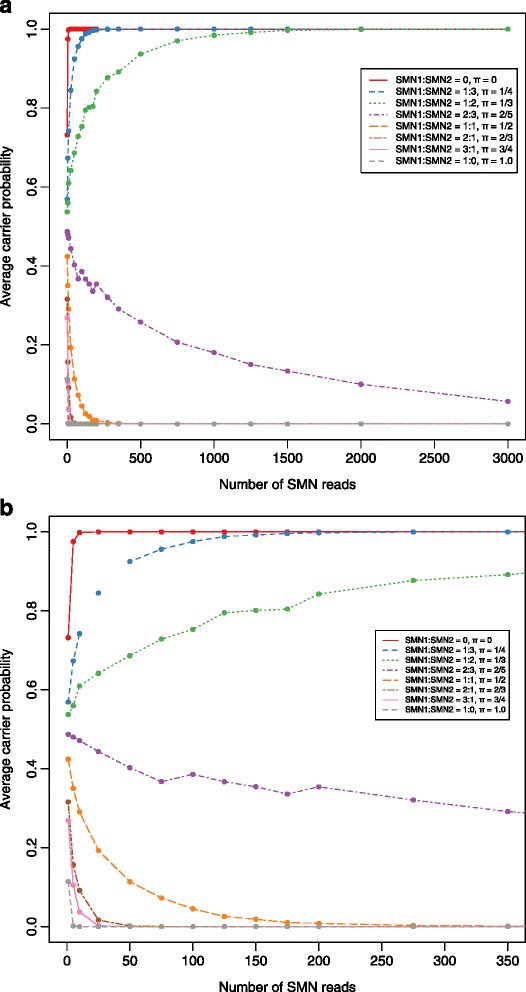
Simulation results of the number of *SMN* reads versus the posterior carrier probability for various levels of π for a maximum of (**a**) 3,000 and (**b**) 350 reads. Each point represents the average across 1,000 permutations. The red, blue, and green lines correspond to values of π less than or equal to 1/3 correspond to true carriers

We simulated 1,000 individuals at three carrier and five non-carrier *SMN1*:*SMN2* copy number ratios (0, 1:3, & 1:2, and 2:3, 1:1, 2:1, 3:1, & 1:0, respectively) with various *SMN* total reads (1 to 3,000) and calculated their average carrier probabilities with our method (Methods). For hypothetical carriers (π ≤ 1/3), a minimum of 350 *SMN* reads are needed to obtain an average carrier probability of 0.90 (i.e., 90 % sensitivity; Fig. 2). However, even with 150 *SMN* reads, the average posterior probability among simulated carriers is at least 0.80 (Fig. 2b). With just 10 reads aligning to our loci of interest in *SMN*, SMA affected individuals (i.e., *SMN1*:*SMN2 =* 0, π = 0) can be assigned an average probability of at least 0.998 for having one or fewer *SMN1* gene copies. Non-carriers with at least a 1:1 *SMN1*:*SMN2* ratio (π ≥ ½) have an average carrier probability less than 0.10 (90 % sensitivity) with more than 75 reads, and 0.001 (99.9 % sensitivity) with more than 200 reads. Simulated individuals without any *SMN2* copies have an average carrier probability less than 10^−5^ with just 10 reads. Non-carrier subjects with a 2:3 *SMN1*:*SMN2* ratio (π = 2/5), require at least 1,000 reads for a carrier probability less than 0.20 (Fig. 2a).

For most subjects (95.8 % in the volunteer and Coriell cohort and 74.3 % in 1KG3 set), we pooled data obtained from all three canonical *SMN1*-defining base positions. However, if the proportion of reads that aligned to *SMN1* at either of our two intronic sites differed from the one in exon 7 by more than 10 %, we calculated our observed proportion based only on the reads aligning to the unique site in exon 7. The three volunteer and 642 1KG3 subjects for whom this is the case have significantly lower coverage in this region of the genome compared to those samples that do not fail the 10 % criterion (*p*-values = 5.46 x 10^−6^ and 3.45 x 10^−8^, respectively).

### Scaled *SMN1* reads

Because the DNA sequences of *SMN1* and *SMN2* are so similar, and their copy numbers so variable, we extended our method to compare the total *SMN* coverage in each sample to that of several “housekeeping” genes. These genes were selected based on their high coverage and low variability in the majority of subjects (Methods). Individuals with a canonical genotype (Fig. 1a) will have one read aligning to *SMN1* and *SMN2* (4 total copies) for every reference gene (2 copies). We account for the number of *SMN* copies with a weighted average of the *SMN* to housekeeping genes ratio (our “scaling factor”), which is capped at 1.00. We multiply the number of reads aligning to our loci of interest in *SMN1* by this scaling factor, and use these values to estimate the scaled probability that a read aligned to this region is actually from *SMN1* (denoted as ${\widehat{\pi}}^{\prime }$).

We examined 20 distinct housekeeping genes in the volunteer, Coriell (Additional file 5: Table S3) and 1KG3 samples (Additional file 6: Table S4). Only three genes (*FASTKD2*, *RAB3GAP1*, and *SLC35D1*) were included on both lists.

The estimate of π for the majority of samples was not drastically affected by the scaling of reads (Additional file 7: Table S5, Additional file 8: Table S6, Additional file 9: Figure S3). About half of the volunteer and Coriell samples (54.9 %) and of 1KG3 samples (52.1 %) have scaling factors not equal to 1.0, with average values of 0.84 (Additional file 7: Table S5, Additional file 8: Table S6). The saliva and semen volunteer samples do not have different average scaling factors (*p*-value = 0.137). The estimates of π before and after scaling are very tightly correlated for both cohorts (Spearman rank correlation ≥ 0.96, *p*-values < 2.2 x 10^−16^). Further, only three volunteer and Coriell samples (A23, C15, and NA03185) and 30 1KG3 samples had carrier probabilities that differed by more than 0.1 due to scaling (Additional file 7: Table S5, Additional file 8: Table S6).

### Sequencing method accurately classifies SMA carriers and non-carriers

The distribution of ${\widehat{\pi}}^{\prime }$ is multimodal among the healthy volunteer and Coriell subjects, with the highest peak near 0.5, corresponding to an equal copy number for *SMN1* and *SMN2* (Additional file 10: Figure S4a). There is no difference in the average ${\widehat{\pi}}^{\prime }$ values for the saliva versus sperm samples (*p*-value = 0.507). The six subjects with values of ${\widehat{\pi}}^{\prime }$ at or near 1.0 have a homozygous loss of *SMN2*, and the five with ${\widehat{\pi}}^{\prime }$ values at or near 0.0 have a homozygous loss of *SMN1* (i.e., they are affected with SMA).

**Fig. 3:**
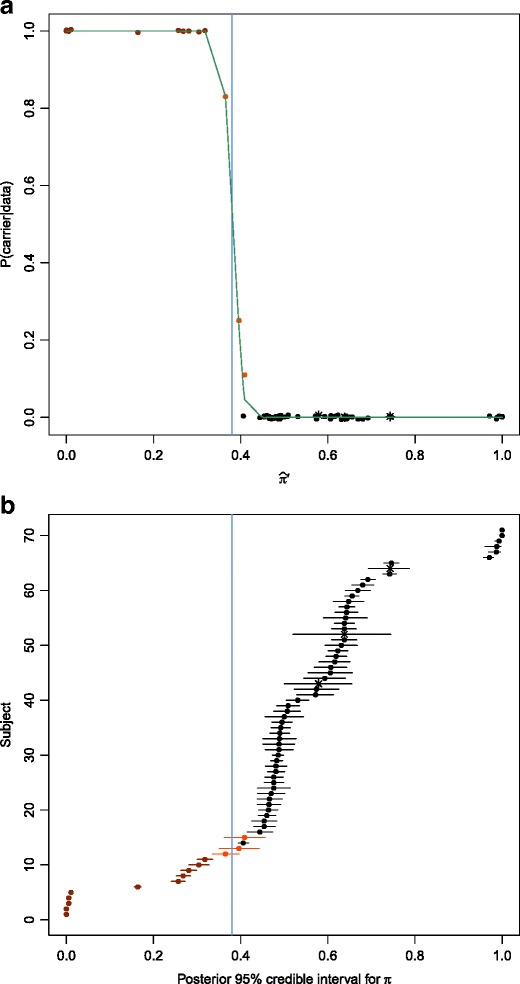
Results for volunteer and Coriell samples. **a**. Estimate of the scaled proportion of *SMN* reads that are from *SMN1* versus the carrier probability for each subject. The carrier probability can be interpreted as the probability that a point on the x-axis falls to the left of the vertical line at 0.38. Samples where few *SMN* reads align to *SMN1* are likely SMA carriers, whereas those with a high proportion of *SMN1* reads are unlikely SMA carriers. **b**. 95 % Posterior (credible) intervals for π are plotted for each subject. In both (a) and (b), subjects that did not meet our 10 % threshold across our three loci are labeled with stars. Note that their intervals in (b) are much wider due to their low coverage. Subjects in both plots are colored red if their credible interval is entirely below the 0.38 cutoff (vertical blue line) and orange if their interval overlaps with 0.38

As expected, the relationship between ${\widehat{\pi}}^{\prime }$ and the posterior probability that π is less than 0.38 given the data (i.e., the carrier probability) has a reverse sigmoidal shape (Fig. 3a). Subjects with low values for ${\widehat{\pi}}^{\prime }$ have very high carrier probabilities, and vise-versa. Individuals with 95 % credible intervals for π that include 0.38 are “possible” carriers, with intermediate posterior probabilities (Fig. 3b). Subjects with high coverage at our SMN gene-defining base positions have very narrow credible intervals (Additional file 7: Table S5).

One Coriell subject (NA03815), who fathered two affected offspring, had an *SMN1* to *SMN2* reads ratio close to $1:1\left(\widehat{\pi}=0.53\right)$. Without scaling, he has a very low carrier probability (2.14 x 10^−92^). However, after scaling, the proportion of his *SMN* reads attributable to *SMN1* is 0.26, resulting in a final carrier probability of 1.0 (Additional file 7: Table S5). A volunteer’s saliva sample (A23) had a similar pattern (initial carrier probability of 9.00 x 10^−10^, final carrier probability of 0.83). The most parsimonious explanation for these two cases is that both individuals have a single *SMN* gene with two recombination-derived alleles (represented as r1 and r3 in Fig. 1d). Thus, in formal genetic terms, these two individuals are heterozygous for a single base mutation in *SMN1* (i.e., *SMN2*).

In addition to NA03815, all of the known SMA carriers and affecteds have a carrier probability of 1.0 according to our computational protocol (Additional file 7: Table S5). The five affected SMA samples have ${\widehat{\pi}}^{\prime }$ values near 0.0, and 95 % credible intervals for π with upper bounds below 0.02 (Additional file 7: Table S5, Fig. 3b). The four unaffected SMA carriers have 95 % credible intervals for π with upper bounds below 0.33. One of our non-SMA diseased control Coriell samples (NA11067), is a male with SMAX1 (OMIM: 313200), an X-linked version of SMA caused by an expansion in *AR* (see [35] for a review); he also has an SMA carrier probability of $1.0\left({\widehat{\pi}}^{\prime }=0.32\right)$. The maximum carrier probability of the five other non-SMA diseased control Coriell samples (who were NHCs or affected with DMD or ALS) is 3.35 X 10^−6^ (Additional file 7: Table S5).

Among the volunteer samples, one (C21) is a clear carrier (${\widehat{\pi}}^{\prime }$ = 0.30, carrier probability = 1.0). Three others (A23, C15, and M21) are possible SMA carriers, with posterior probabilities above 0.10 (Additional file 7: Table S5).

### Sequencing carrier status replicated by MLPA

We used MLPA to determine the carrier status for a subset of our volunteer and Coriell samples. Of the 24 volunteer samples that were not chosen for MLPA, eight had insufficient DNA quantities (≤495ng of DNA, a minimum of 600ng was needed for MLPA) after using these samples for DNA-seq. We are confident that an additional 16 individuals have at least two copies of *SMN1* due to their high coverage (average *SMN* reads = 1352.13, range = [372, 2764]) and values for ${\widehat{\pi}}^{\prime }$ (average ${\widehat{\pi}}^{\prime }$ = 0.539, range = [0.460, 0.680]) which led to their exclusion from the MLPA analysis. None of these samples were likely carriers (carrier probability ≤ 2.86 X10^−8^; Additional file 7: Table S5).

**Fig. 4:**
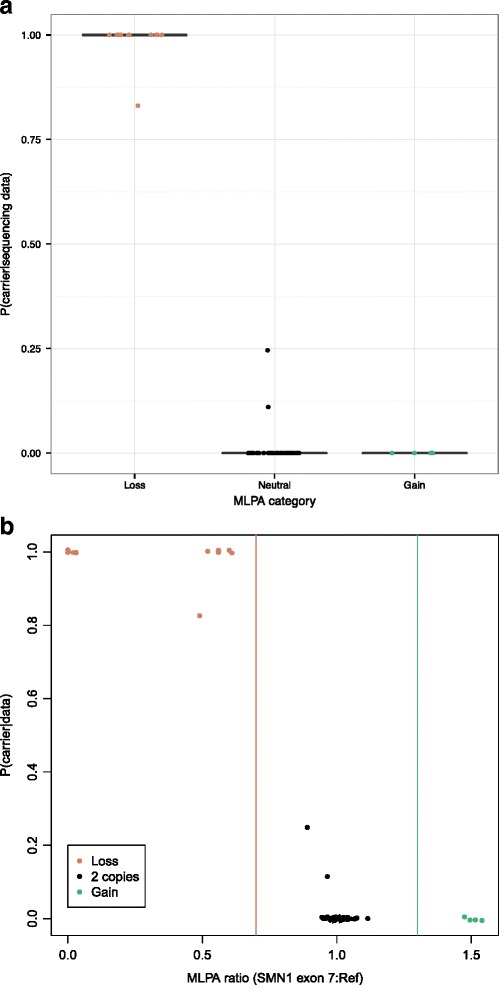
Results from the MLPA analysis in 60 subjects. **a**. Carrier probability for each sample stratified by MLPA category. **b**. Ratio of *SMN1* exon 7 to reference sample versus carrier probability for each sample. Vertical lines at 0.7 and 1.3 represent thresholds for copy number loss and gain, respectively. In both (a) and (b), tan, black, and green points represent samples that have less than, equal, and more than two copies of *SMN1* exon 7, respectively

We found a strong correlation between the sequencing-based and MLPA carrier statuses for each sample (Fig. 4, Additional file 7: Table S5). Eleven of the twelve samples that are carriers by the MLPA method (*SMN1*:reference < 0.7) have a carrier probability of 1.0, and the twelfth sample (A23) has a very high carrier probability (0.83) based on our sequencing method (Additional file 7: Table S5; Fig. 4). Further, only two of the 35 non-carriers (MLPA *SMN1*:reference > 0.7) have carrier probabilities above 7.51 X 10^−6^ based on their sequencing data; these two have low posterior probabilities of 0.25 (C15) and 0.11 (M21) (Additional file 7: Table S5; Fig. 4). The SMAX1 Coriell subject (NA11067) is a carrier based on his MLPA result (*SMN1*:reference = 0.52).

### Proportion of 1KG3 carriers closely matches corresponding population

Consistent with the published frequencies for *SMN1* and *SMN2* copy numbers [15, 17] and the results for the volunteer and Coriell subjects, the multimodal distribution of the ratio of *SMN1* to *SMN* reads has peaks near the three most common genotypes: 0.50 (corresponding to a 2:2 *SMN1*:*SMN2* ratio), 0.67 (2:1 *SMN1*:*SMN2* ratio), and 1.0 (2:0, 3:0, or 4:0 *SMN1*:*SMN2* ratio) (Additional file 10: Figure S4b).

**Fig. 5:**
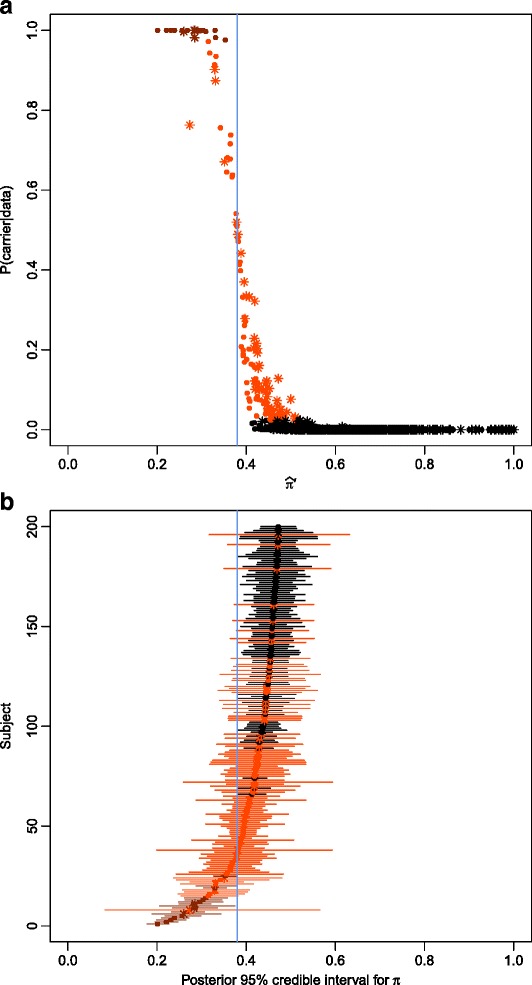
Results for 1000 Genomes samples. **a**. Estimate of the scaled proportion of *SMN* reads that are from *SMN1* versus the carrier probability for each subject. **b**. 95 % Posterior (credible) intervals for π are plotted for subjects with the lowest 200 estimates of π. In both (a) and (b), subjects are colored and labeled under the same criteria as in Fig. 3

Based on our ternary groupings, there are 16 1KG3 subjects who are high-probability carriers, 109 possible carriers, and 2,376 unlikely carriers (Fig. 5, Additional file 8: Table S6, Additional file 11: Figure S5). The possible carriers had significantly lower coverage than the other 2,392 individuals in this cohort (average total coverage of 248.0 and 486.9, respectively, *p*-value = 9.59 X 10 ^−15^).

In order to compare the carrier frequency in five 1KG3 superpopulations to those from a recent large meta-analysis [14], we reclassified all subjects into binary “carrier” and “non-carrier” divisions. We assigned all subjects who are more likely than not to be carriers as “carriers” (posterior probability >0.5), and all others as “non-carriers”. Even though some of these subjects have lower than optimal coverage, their posterior probabilities take into account this low coverage and corresponding uncertainty are thus still interpretable.

We found no statistical difference (two-sided exact binomial test, *p*-value ≥ 0.10) for any population, with the exception of the “Asian Indian” population (*p*-value = 0.02) (Table 1). However, the Asian Indian frequency was determined from a single study of just 976 individuals [19], where American subjects self-categorized into one of six ethnic groups. Thus, this Asian Indian carrier frequency truly represents self-identified Americans of Indian descent, not a random sample of individuals from the Indian subcontinent. Each of our exact confidence intervals for the carrier frequencies, including the Asian Indian subjects, overlapped with the published intervals.

**Table 1:** Carrier frequencies for select super-populations in the 1000 Genomes dataset

Superpopulation (subpopulations)	Number of Samples	Number of Carriers (P(carrier) > 1/2)	Carrier frequency, p_Obs_ (95 % exact CI)	Published carrier frequency, p_0_ (95 % CI)	*P*-value
					(H_0_: p_Obs_ = p_0_)
Caucasian (CEU, FIN, GBR, IBS, TSI)	503	13	0.026 (0.014 , 0.044)	0.022 (0.020, 0.024)	0.543
Black (ACB, ASW, ESN, GWD, LWK, MSL, YRI)	661	4	0.006 (0.002, 0.015)	0.014 (0.009, 0.018)	0.095
Asian (CDX, CHB, CHS, JPT, KHV)	502	6	0.012 (0.004, 0.026)	0.020 (0.016, 0.024)	0.261
Hispanic (MXL, PUR, CLM)	261	6	0.023 (0.009, 0.049)	0.012 (0.006, 0.017)	0.140
Asian Indian (GIH, ITU, STU, BEB, PJL)	489	3	0.006 (0.001, 0.018)	0.020 (0.010, 0.029)	0.022
All	2501	36	0.01 (0.010, 0.020)	0.019 (0.018, 0.020)	0.138

## Discussion

We developed and validated a computational protocol to perform SMA carrier screening on individual exome sequence data. Our protocol eliminates the need to test for SMA in a process distinct from all other carrier tests that can be multiplexed in a single targeted gene panel. Previous sequencing methods were restricted by their inability to differentiate between the *SMN1* and *SMN2* gene paralogs [15]. We overcome this limitation by focusing on three nucleotide positions that are unique to each gene.

We apply a Bayesian model to compute the probability that a given individual is an SMA carrier given his or her sequencing data at these loci. For each subject, we measure his or her actual carrier probability on a continuous scale. In this way, we have a precise quantification of carrier risk, which can lead to a more specific prediction of affected progeny for any given couple.

We tested our technique on healthy volunteers and samples with known carrier status from the Coriell repository. Our sequencing and qPCR based results for these samples led to identical conclusions; all likely carriers (or non-carriers) by one method were carriers (or not) by the other. We analyzed two carriers who have a 1:1 ratio of *SMN1* to *SMN2* reads. These individuals have one copy each of *SMN1* and *SMN2*, yet their *SMN1*:*SMN2* reads ratio is no different than if they had two copies each of *SMN1* and *SMN2* (or three, four, etc.) and were not carriers. Nevertheless, we calculated a high carrier probability for both of these subjects because our method scales *SMN1* reads relative to several “housekeeping” genes.

We also identified a carrier among our non-SMA diseased control samples. This subject has been diagnosed with SMAX1, and is not listed as an SMA carrier in the Coriell database. However, according to both our method and the MLPA analysis, he is an SMA carrier. This is a crucial characteristic and should be added to his public profile. Because he is an SMA carrier, there is some chance that his SMAX1 affected family members have been misdiagnosed and may actually have SMA.

To demonstrate the utility of our method, we determined the carrier status of 2,501 subjects from the Phase 3 release of the 1000 Genomes Project based on only their available sequencing data. These individuals represent a diverse global sample of adults without severe pediatric disease, and their SMA carrier status was previously unknown. The majority of these samples are not readily available for a wet lab carrier detection method; a DNA-seq method is the only way to determine their SMA carrier status. Our method is able to assign all subjects in this cohort a carrier probability from sequencing data alone, regardless of coverage. This is one of the advantages of our method and a continuous carrier probability outcome. The sequencing-based carrier rates in the 1KG3 data were no different from the consolidation of several PCR-based experiments.

The coverage of *SMN* necessary to accurately determine carrier status is dependent upon the underlying genotype of a given individual. For use of our method in the clinic, we recommend a at least 350 *SMN* reads for 90 % sensitivity, with the caveat that 2:3 *SMN1*:*SMN2* subjects may still have intermediate carrier probabilities at this coverage. These individuals are relatively rare (1-3 % of the general population) [16, 17], and have an average carrier probability of only 0.295 at our recommended coverage level based on our simulation.

Because our carrier probabilities and corresponding credible intervals for π incorporate the uncertainty associated with low coverage, samples with fewer than 350 *SMN* reads can still be analyzed by our method. These low coverage samples will have carrier probabilities closer to ½ (i.e., more uncertainty) and wider intervals than if they had more reads, but for many genotypes, this will not result in the reclassification of a sample. In our analysis, four (5.6 %) of the volunteer and Coriell samples and 1,085 (43.4 %) of the 1KG3 samples (including 91 of 109 of the “possible” carriers) did not meet the recommended coverage. For the purpose of quantifying the carrier status of all subjects given only the provided data at hand, we included everyone in our analysis, regardless of coverage.

As with existing SMA carrier detection methods, our approach cannot take into account haplotype phase nor identify *cis*, or silent “2 + 0”, carriers (i.e., individuals with at least two copies of *SMN1* on one chr5, but no *SMN1* the other), without additional information [21]. About 2-5 % of SMA carriers and about 1/800 individuals have the 2 + 0 genotype [36–38]. Our method could be extended to utilize distinct population polymorphisms to detect these carriers (e.g., in Ashkenazi Jews [39]). Further, neither our method nor existing methods can be used to prevent progeny with *de novo* mutations, which occur in approximately 2 % of SMA cases [10, 40].

## Conclusions

DNA sequencing has become the preferred tool for recognizing disease-causing variants throughout the genome. Large, multi-gene targeted sequencing panels are the future of clinical carrier testing [41], and there is a critical need for a comprehensive NGS test that includes SMA carrier detection. The protocol presented here should be implemented as part of broad genetic sequencing screening tests to concurrently assess carrier risk for multiple Mendelian diseases with only NGS data. Without the extra step of qPCR, our approach is the sole process that exclusively utilizes DNA-seq output to measure the likelihood of an SMA carrier.

## Availability of supporting data

Read counts at the loci of interest in *SMN1* and *SMN2* for all volunteer samples can be found in Additional file 7: Table S5.

## Additional files

Additional file 1: Figure S1. (a) Cartoon schema of differences between *SMN1* and *SMN2*. Loci of interest are highlighted in red. (b) An overview of the method presented. (PDF 379 kb) Additional file 2: Table S1. MLPA probes details. (XLS 25 kb) Additional file 3: Table S2. Theoretical results. (XLSX 39 kb) Additional file 4: Figure S2. Posterior carrier probabilities for each volunteer sample under a uniform (x-axis) and under Jeffreys (y-axis) prior. Samples are colored and symbolized as in Fig. 3. (PDF 151 kb) Additional file 5: Table S3. Housekeeping genes used for volunteer and Coriell samples. (PDF 5 kb) Additional file 6: Table S4. Housekeeping genes used for 1000 Genomes Project samples. (XLSX 39 kb) Additional file 7: Table S5. Results for all volunteer and Coriell samples. (XLSX 49 kb) Additional file 8: Table S6. Results for all 1000 Genomes Project samples. (XLSX 266 kb) Additional file 9: Figure S3. A plot of the proportion of reads aligning to *SMN1* for each volunteer and Coriell subject when using the raw reads (x-axis) versus those calculated from scaling the reads based on housekeeping ratios (y-axis). Subjects shaped as stars did not meet the ε criteria; they have a high level of variability across all three sites. All subjects either have the same value for both scaled and unscaled π (i.e., they fall exactly on the 45 ° line), or they have smaller scaled π values (below the line). Individuals whose scaled estimate of π is below the horizontal blue line at 0.38 are likely carriers. Subjects colored in red are likely carriers; their posterior intervals are entirely below our 0.38 cutoff. The intervals of orange colored subjects overlap with 0.38; these subjects are possible carriers. (PDF 91 kb) Additional file 10: Figure S4. A plot of the scaled proportion of reads aligning to *SMN1* versus their frequency for (a) the volunteer and Coriell subjects and (b) the 1000 Genomes subjects. In both datasets, most individuals have an estimate of π to the right of the line at 0.38; it is unlikely they are carriers. (PDF 114 kb) Additional file 11: Figure S5. 95 % Posterior (credible) intervals for π are plotted for each 1000 Genomes Project subject. Samples are colored and symbolized as in Fig. 5. (PDF 62 kb)

## Abbreviations

1KG3: 1000 Genomes Project Phase 3
ALS: amyotrophic lateral sclerosis
DMD: Duchenne muscular dystrophy
MLPA: multiplex ligation-dependent probe amplification
NHC: normal healthy control
qPCR: quantitative polymerase chain reaction
SMA: spinal muscular atrophy
SMAX1: X-linked SMA
