# Supplementary figures and images for: Validation of a high resolution NGS method for detecting spinal muscular atrophy carriers among phase 3 participants in the 1000 Genomes Project
Source: BMC Med Genet. 2015 Oct 29;16:100. doi: 10.1186/s12881-015-0246-2 (PMC4625734; doi:10.1186/s12881-015-0246-2)

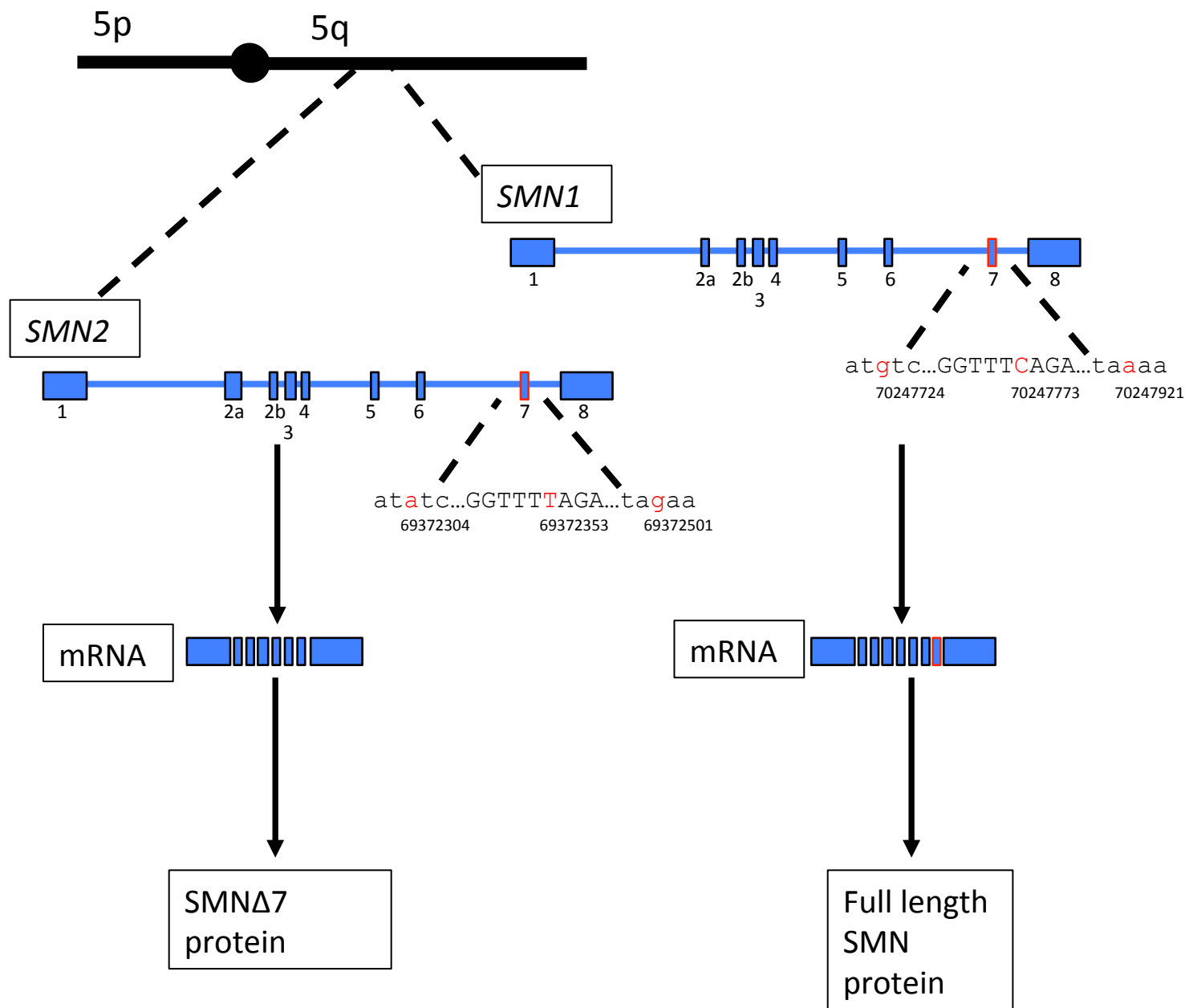

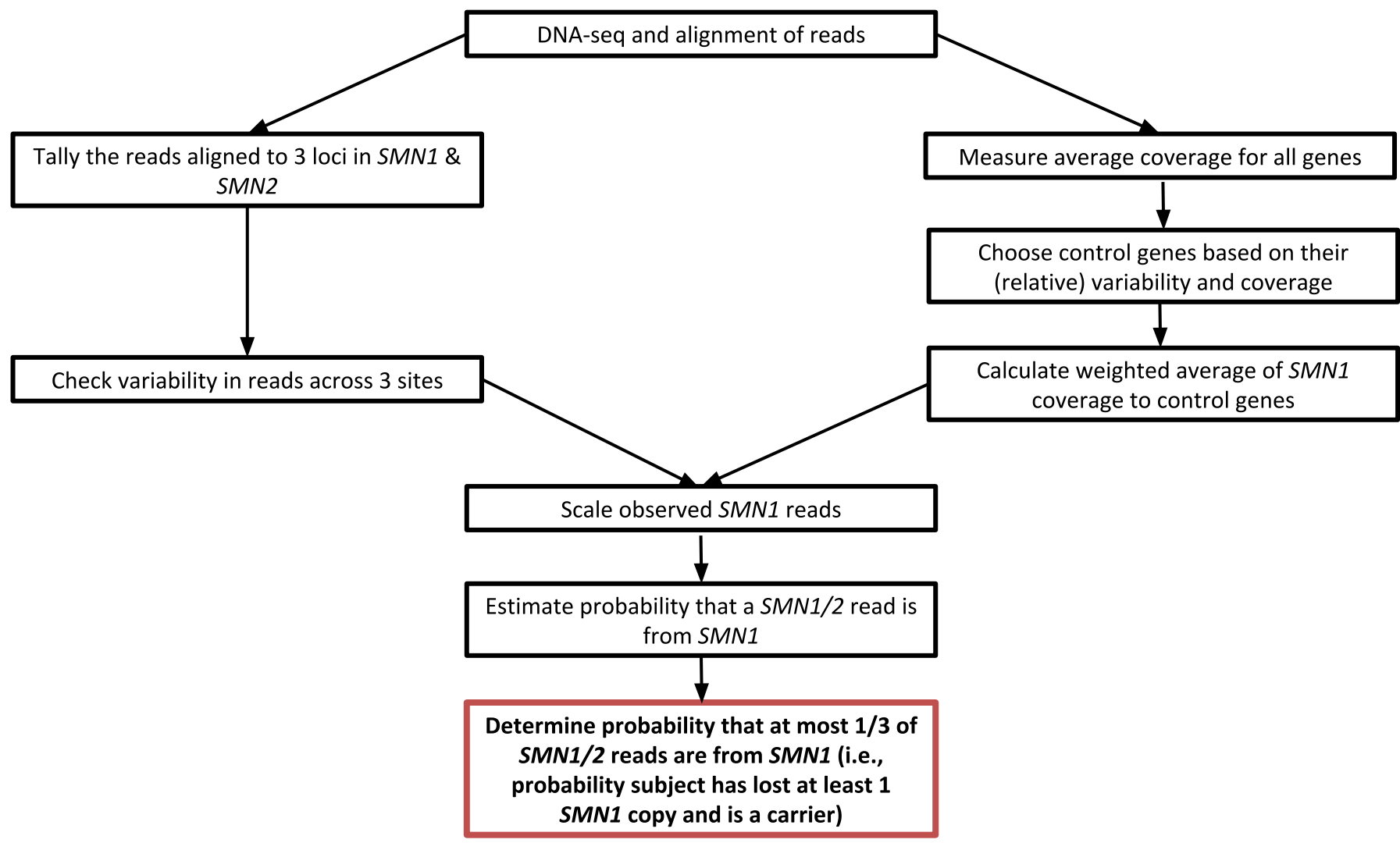

Supplement: Additional file 1: Figure S1. — (a) Cartoon schema of differences between SMN1 and SMN2. Loci of interest are highlighted in red. (b) An overview of the method presented. (PDF 379 kb) [file 12881_2015_246_MOESM1_ESM.pdf]

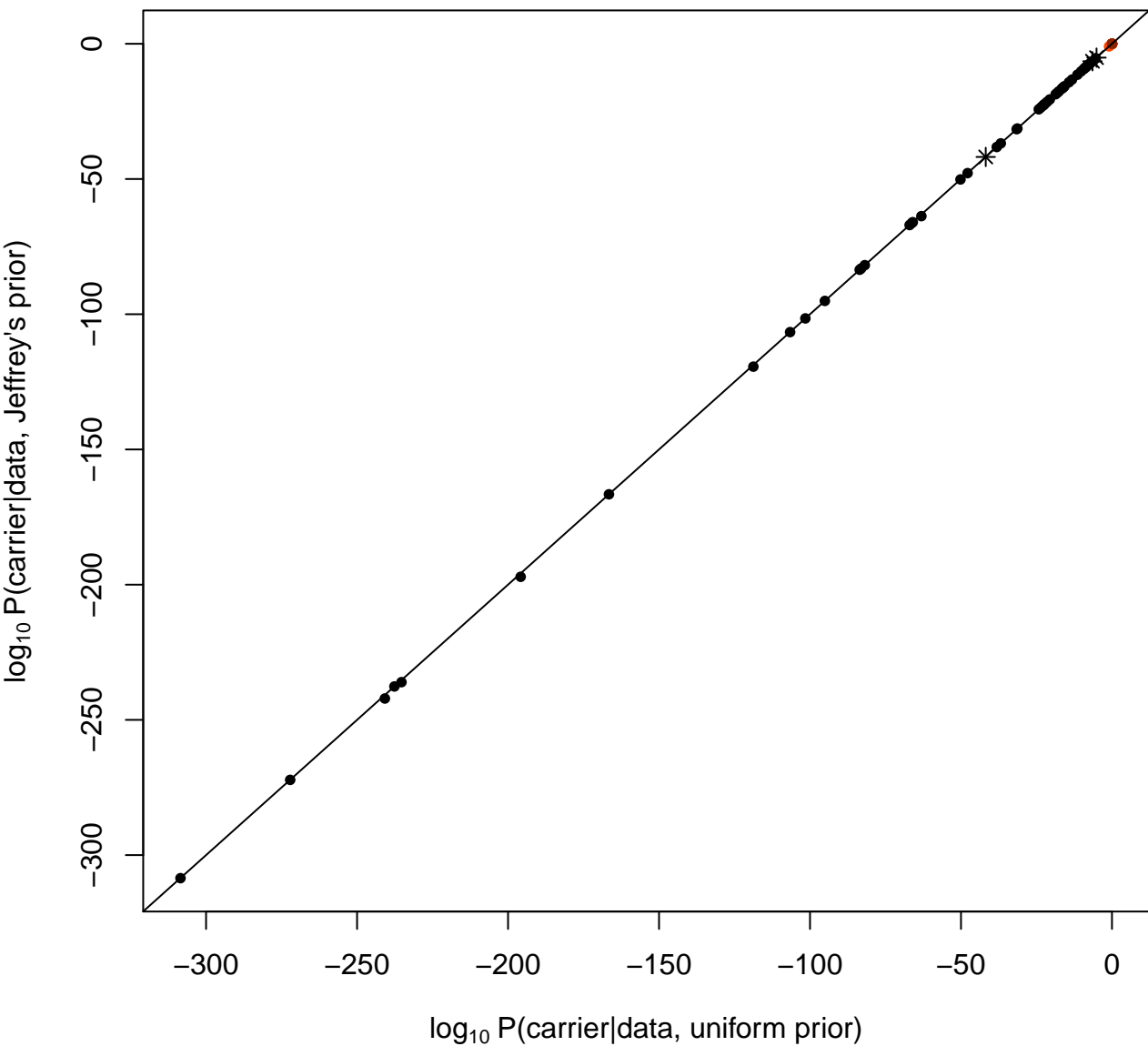

Supplement: Additional file 5: Table S3. — Housekeeping genes used for volunteer and Coriell samples. (PDF 5 kb) [file 12881_2015_246_MOESM5_ESM.pdf]

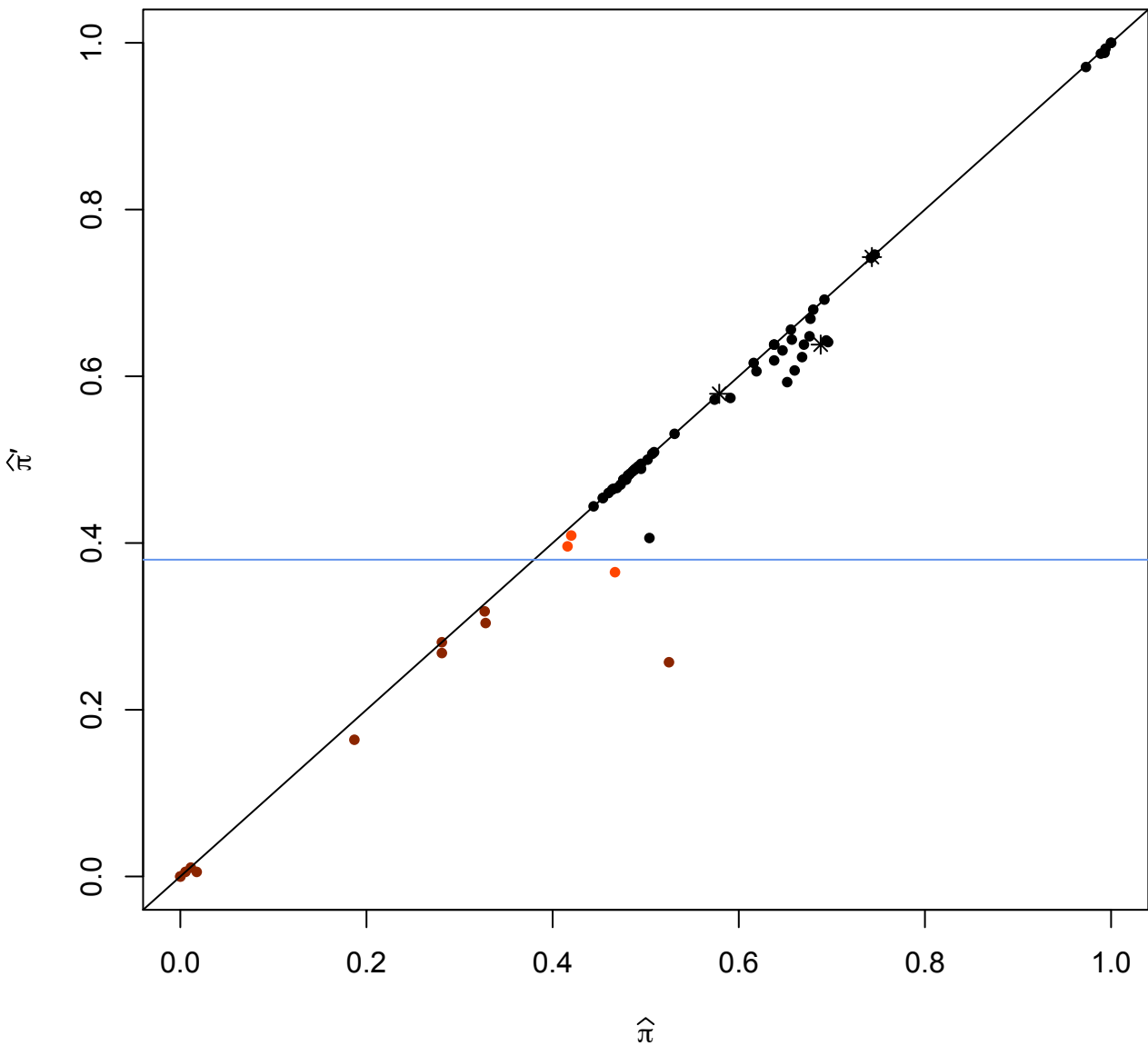

Supplement: Additional file 9: Figure S3. — A plot of the proportion of reads aligning to SMN1 for each volunteer and Coriell subject when using the raw reads (x-axis) versus those calculated from scaling the reads based on housekeeping ratios (y-axis). Subjects shaped as stars did not meet the ε criteria; they have a high level of variability across all three sites. All subjects either have the same value for both scaled and unscaled π (i.e., they fall exactly on the 45 ° line), or they have smaller scaled π values (below the line). Individuals whose scaled estimate of π is below the horizontal blue line at 0.38 are likely carriers. Subjects colored in red are likely carriers; their posterior intervals are entirely below our 0.38 cutoff. The intervals of orange colored subjects overlap with 0.38; these subjects are possible carriers. (PDF 91 kb) [file 12881_2015_246_MOESM9_ESM.pdf]

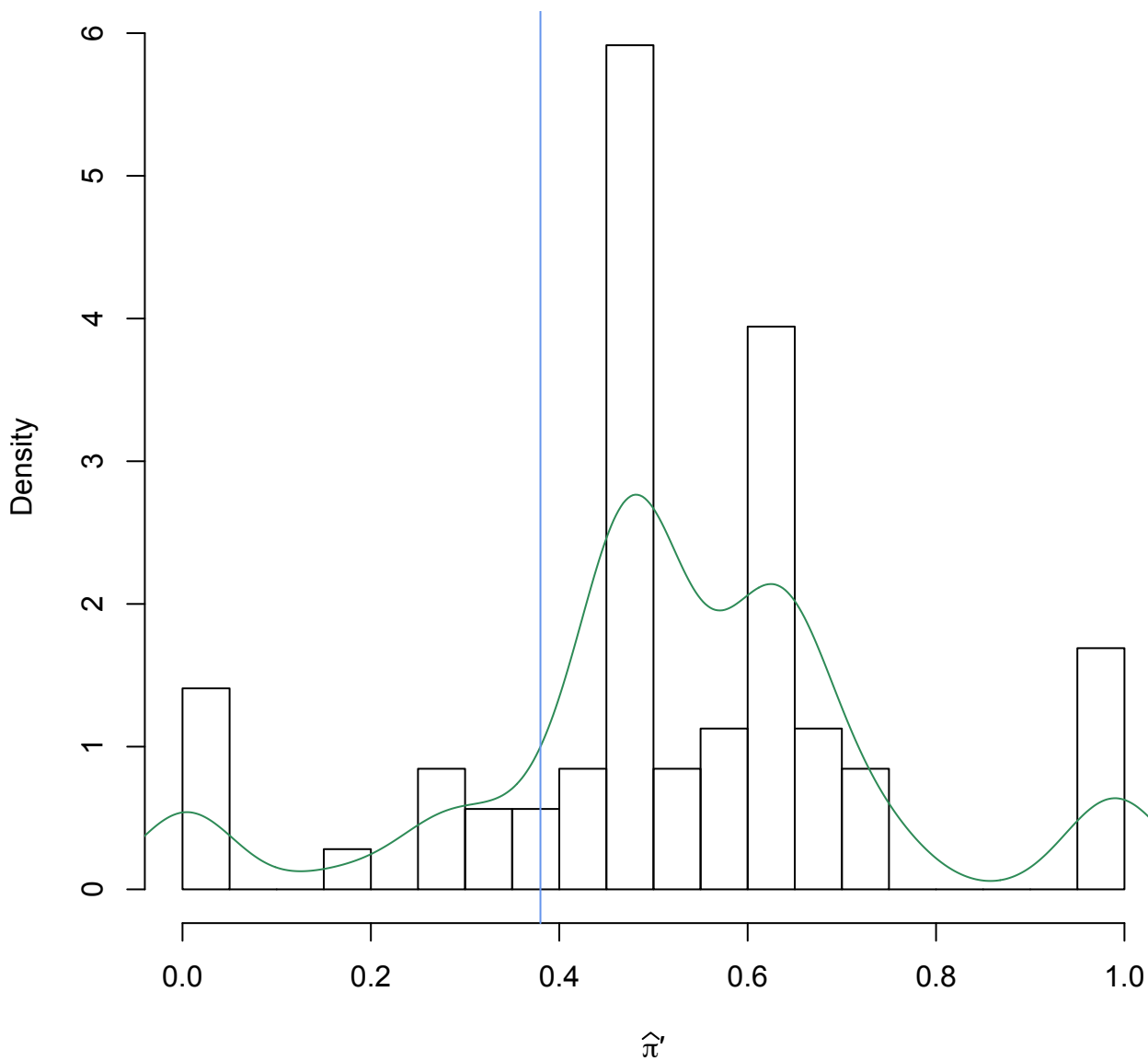

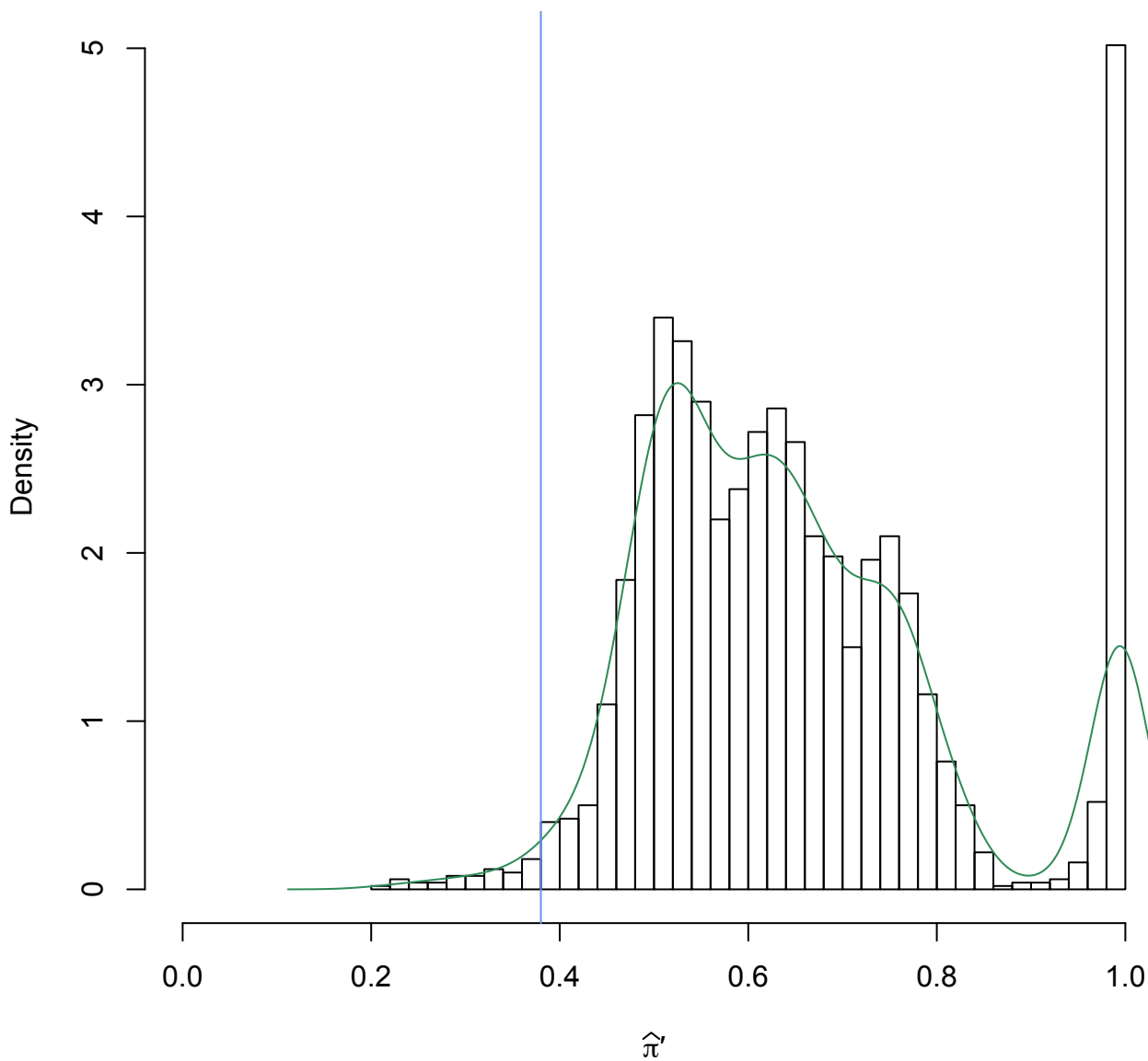

Supplement: Additional file 10: Figure S4. — A plot of the scaled proportion of reads aligning to SMN1 versus their frequency for (a) the volunteer and Coriell subjects and (b) the 1000 Genomes subjects. In both datasets, most individuals have an estimate of π to the right of the line at 0.38; it is unlikely they are carriers. (PDF 114 kb) [file 12881_2015_246_MOESM10_ESM.pdf]

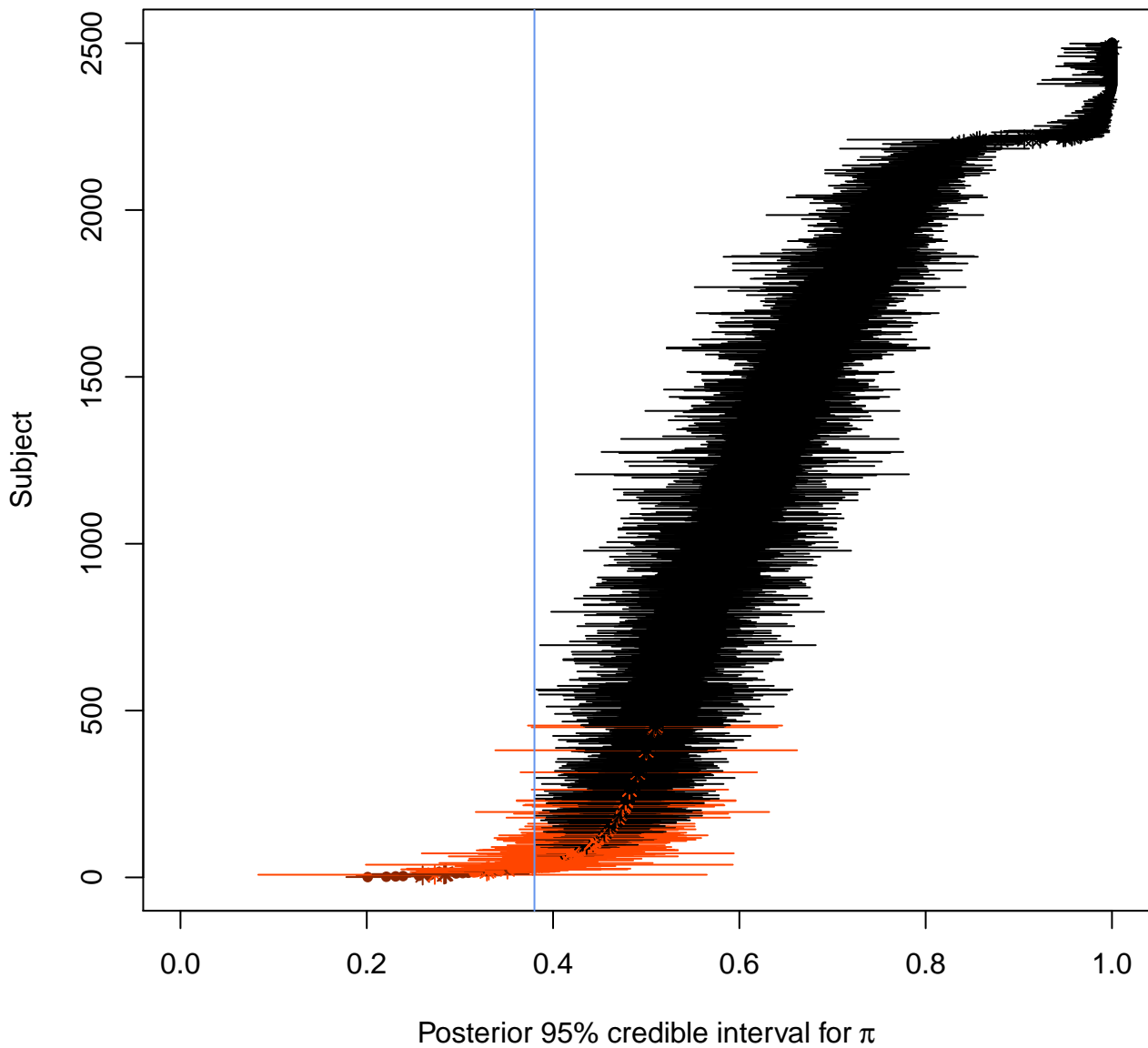

Supplement: Additional file 11: Figure S5. — 95 % Posterior (credible) intervals for π are plotted for each 1000 Genomes Project subject. Samples are colored and symbolized as in Fig. 5. (PDF 62 kb) [file 12881_2015_246_MOESM11_ESM.pdf]
