# Supplementary material for: Validation of a high resolution NGS method for detecting spinal muscular atrophy carriers among phase 3 participants in the 1000 Genomes Project
Source: BMC Med Genet. 2015 Oct 29;16:100. doi: 10.1186/s12881-015-0246-2 (PMC4625734; doi:10.1186/s12881-015-0246-2)
Supplement: Additional file 4: Figure S2. — Posterior carrier probabilities for each volunteer sample under a uniform (x-axis) and under Jeffreys (y-axis) prior. Samples are colored and symbolized as in Fig. 3. (PDF 151 kb) [file 12881_2015_246_MOESM4_ESM.pdf]

SMN1 copy number	*SMN2* copy number	$\hat{\theta}$	Population frequency (%)	D	D'	r	$\frac{D'}{r} = \hat{\pi}'$	Posterior Likelihood $P(\pi \leq 1/3 \mid D', r)$ Conclusion
1	0	0.25	< 1	100	25	100	0.25	Likely carrier
1	1	0.50	< 1	100	50	200	0.25	Likely carrier
2	0	0.50	$5 - 6$	200	100	200	0.50	Unlikely carrier
1	2	0.75	$1 - 2$	100	75	300	0.25	Likely carrier
2	1	0.75	$30 - 40$	200	150	300	0.50	Unlikely carrier
1	3	1.00	< 1	100	100	400	0.25	Likely carrier
2	2	1.00	$45 - 50$	200	200	400	0.50	Unlikely carrier
3	1	1.00	5	300	300	400	0.75	Unlikely carrier
4	0	1.00	< 1	400	400	400	1.00	Unlikely carrier
1	4	1.00	< 1	100	100	500	0.20	Likely carrier
2	3	1.00	$1 - 3$	200	200	500	0.40	Likely/unlikely carrier
3	2	1.00	$1 - 2$	300	300	500	0.60	Unlikely carrier
4	1	1.00	< 1	400	400	500	0.80	Unlikely carrier
